# Predicting Strut Geometry of PCL and DMSO_2_ Biocomposites from Nozzle to Deposition in Bio-Scaffold 3D Printing

**DOI:** 10.3390/ma18102380

**Published:** 2025-05-20

**Authors:** Jae-Won Jang, Kyung-Eun Min, Jun-Hee Park, Cheolhee Kim, Sung Yi

**Affiliations:** Department of Mechanical and Material Engineering, Portland State University, Portland, OR 97201, USA; jaewon@pdx.edu (J.-W.J.); min3@pdx.edu (K.-E.M.); junheep@pdx.edu (J.-H.P.)

**Keywords:** PCL, DMSO_2_, predicting 3D printing geometry, rheology, viscosity, biomaterial, scaffold

## Abstract

The field of tissue engineering increasingly demands accurate predictive models to optimize the 3D printing process of bio-scaffolds. This study presents a unified numerical model that predicts extrusion velocity and strut diameter based on printing conditions and the material properties of polycaprolactone (PCL) and dimethyl sulfone (DMSO_2_) composites. The extrusion velocity was simulated using Navier–Stokes equations, while the strut diameter was calculated via a surface energy model. For PCL, the extrusion velocity showed a temperature coefficient of 23.3%/°C and a pressure coefficient of 19.1% per 100 kPa; the strut diameter exhibited a temperature coefficient of 21.6%/°C and a pressure coefficient of 16.6% per 100 kPa. When blended with DMSO_2_, the lower viscosity and higher surface energy resulted in increased extrusion velocity and strut diameter. The proposed model achieved a high predictive accuracy, with determination coefficient (R²) values exceeding 0.95. These results demonstrate the model’s potential to optimize 3D printing parameters, guide biomaterial selection, and predict pore characteristics, ultimately supporting the rational design of tissue engineering scaffolds.

## 1. Introduction

Bio-scaffolds play a crucial role in tissue engineering by providing a structural framework for cell growth and tissue regeneration [1,2,3]. These bio-scaffolds should require biocompatibility, biodegradability, and sufficient mechanical strength to support cell proliferation and facilitate tissue formation [4,5,6]. In recent years, three-dimensional (3D) printing has attracted considerable attention due to its ability to create intricate structures with high precision and repeatability with excellent shape controllability [7]. In particular, recent developments in multiscale and multimaterial additive manufacturing have expanded the functional scope of 3D printing, enabling advanced structural and compositional designs [8]. In the 3D printing process, achieving optimal printing conditions, such as temperature and pressure, is vital to accurately predict printed 3D scaffold geometry including pore size and porosity [9,10]. Moreover, scaffold geometry plays a vital role in biological performance, as parameters such as pore interconnectivity and size directly influence cell migration, nutrient diffusion, and tissue integration [11].

The utilization of synthetic polymers in bio-scaffold fabrication through 3D printing has been considered widely interesting due to their adjustable mechanical properties as well as biodegradation rate [12]. Among recent advances, Ganguly and Tang [13] demonstrated a solvent exchange postprocessing strategy to fabricate nanocomposites with enhanced thermal and mechanical stability using sustainable materials, which can also offer benefits in developing biocompatible and eco-friendly scaffolds for biomedical applications. Polycaprolactone (PCL) is a well-known synthetic biodegradable polymer that has received approval from the Food and Drug Administration (FDA) as a biopolymer [14]. PCL exhibits a low melting temperature and good thermal stability and is a cost-effective material [15]. Meanwhile, PCL is characterized by a slow degradation rate and low cell attraction [16]. Additionally, high viscosity of PCL requires high printing pressure, resulting in challenges in achieving high dimensional accuracy in 3D printing [17]. Overcoming the limitation of a single biomaterial like PCL, biocomposites including PCL-based composites have been studied in recent years [18,19,20,21]. Especially, Dimethyl sulfone (DMSO_2_) is recently introduced as an additive material in the PCL matrix. PCL and DMSO_2_ composites demonstrate enhanced hydrophilicity, mechanical properties, and the ability to tailor degradation rate compared to pure PCL [22,23].

The biomaterial printability is defined as the ability to fabricate a bio-scaffold using the 3D printing method [24]. Several factors can influence the printability of 3D bio-scaffold including material properties of selected biomaterial and 3D printing parameters such as temperature, pressure, extrusion velocity, and nozzle geometry [9]. The evaluation of printability in bio-scaffold fabrication is an essential aspect as it directly determines the achievement of the desired scaffold structure [25,26,27]. Ouyang et al. [25] defined printability based on the pore shape formed by filament interconnection. They used gelatin/alginate bioink and measured the printability by gelatin concentrations. A composite with 7.5% gelatin demonstrated suitable printability, while lower and higher gelatin concentrations required specific printing conditions, such as low printing temperature and short holding time, to achieve acceptable printability. He et al. [26] investigated the printability of hydrogels for the printing parameters. The evaluation included the dimensional accuracy of printed structures, ranging from one-dimensional to three-dimensional designs. The printing pressure, nozzle feed rate, and layer height were highlighted as the most affecting factors for the printing quality.

The entire 3D printing process through the material extrusion method can be divided into the following steps: material melting within the nozzle, extrusion from the nozzle, molten material transfer from the nozzle to the substrate, and stacking layer by layer on the substrate. Predicting the extrusion velocity and strut diameter is the first step in enhancing the printability of the bio-scaffold [17]. Consequently, various numerical models have been proposed to predict the flow process in material extrusion-based 3D printing [28,29]. Additionally, a machine-learning-based prediction model was recently proposed to replace the experimental-based prediction model [30,31]. Serdeczny et al. [32] conducted computational fluid dynamics (CFD) simulations of the polymer within a hot nozzle. The model predicted a recirculation region between the nozzle wall and the inserted filament, allowing for analysis of pressure and temperature inside the extrusion channel. They figured out the influence of the temperature and nozzle diameter on the flow phenomena. Gosset et al. [33] developed a CFD model simulating the cross-section of printed strut shapes and dimensions. The simulation was well fitted compared to the experimental data, and this model allowed the accurate determination of the input printing conditions. However, these models only focused on one step of the extrusion or stacking behaviors, not considering the entire printing process. Although Lee et al. [34] tried to solve an analytical unified model to predict extrusion velocity and strut diameter, resulting in predicting the geometric characteristics of the 3D bio-scaffold, the assumption that the printed strut had a circular shape was still limited. Hence, developing a unified model capable of accurately predicting the entire process of 3D bio-scaffold fabrication is a significant current discussion in the tissue engineering field.

The objective of this study is to introduce a unified numerical model that can effectively predict the extrusion velocity and strut diameter in the entire 3D printing process, taking into consideration the material properties and printing conditions. The results from the developed model were compared to the experimental data to verify accuracy. The printing parameters of the printing conditions and material properties were discussed to evaluate the effect on the extrusion velocity and strut diameter. This study aims to enhance the understanding of the relationship between the printing parameters and the results in the 3D printing process through the development and validation of this numerical model.

## 2. Materials and Methods

### 2.1. Materials

This study investigated the effects of DMSO_2_ on the material extrusion and stacking models compared to PCL and PCL-based composites. Powdered PCL with a molecular weight of 50000 was obtained from Polysciences (Warrington, PA, USA), while powdered DMSO_2_ was obtained from Bergstrom Nutrition (Vancouver, WA, USA). The material properties of both materials are listed in Table 1. During the feasibility test, the composites exceeding 40 wt% of DMSO_2_ concentrations formed too brittle material to keep the 3D structure. Composites with DMSO_2_ concentrations of 10, 20, and 30 wt% based on the PCL matrix were prepared using a physical mixing process with an electric milling machine (YUESUO, Zhengzhou, China). All composites were dried at 45 °C under vacuum conditions for one day before mixing. In this study, the composites were labeled as PCL/D10, PCL/D20, and PCL/D30 according to their respective DMSO_2_ weight percentages.

### 2.2. Printing Equipment and Process

A pneumatic material extrusion 3D printer, Allevi 2 (Allevi, Inc., Philadelphia, PA, USA), was used to print out the materials. Input parameters of the 3D printer were the printing temperature and pressure ranging from room temperature to 140 °C and 6.90–827 kPa, individually. The printing conditions were 120–140 °C of printing temperature with an interval of 5 °C and 138–621 kPa of pressure with an interval of 68.9 kPa. A nozzle inner diameter of 450 um and nozzle moving speed of 0.8 mm/s were fixed to focus on the effects of printing temperature and pressure as well as to evaluate the effect of material properties for extrusion velocity and strut diameter. The extrusion behavior was recorded using an EOS REBEL T3i DSLR camera (CANON, Tokyo, Japan), and the recorded videos were cut into images with 30 frames per second to calculate the extrusion velocity for each printing condition. The straight line with 2 mm of length was printed to measure the printed strut diameter at all printing conditions.

### 2.3. Modeling

**Figure 1:**
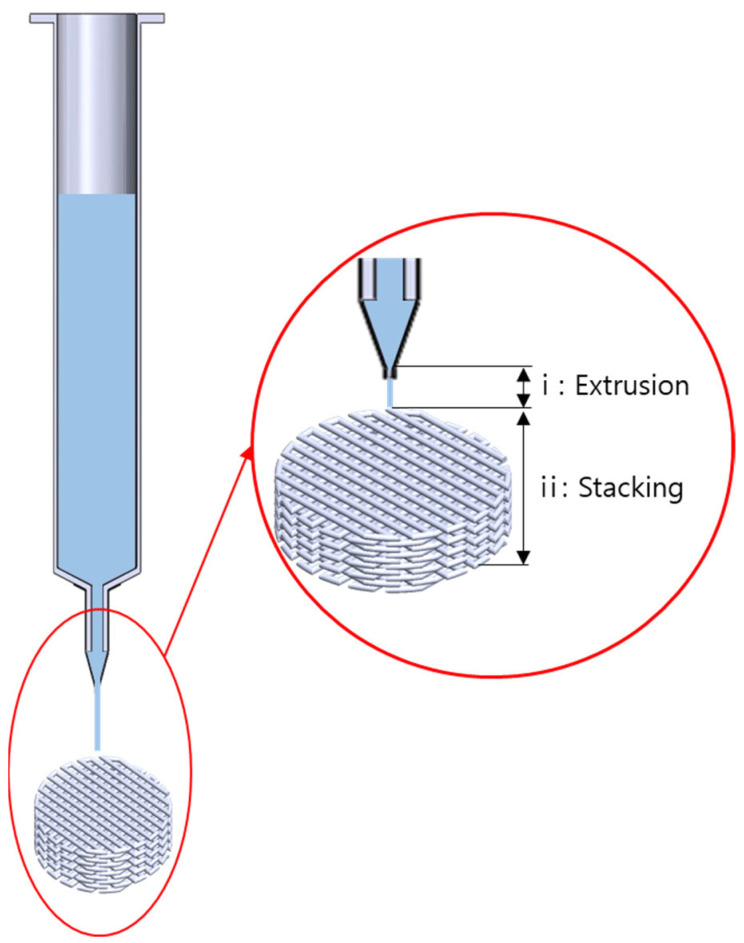
Schematic view of the printing process system.

In this study, the extrusion and stacking models were considered (Figure 1) for predicting strut geometry. The extrusion velocity from the extrusion model was put into the input of the stacking model.

#### 2.3.1. Viscosity Model

The flow field for incompressible fluid can be calculated by the Navier–Stokes equations (Equation (1)),

(1) $$\frac{\partial u}{\partial x}+\frac{\partial v}{\partial y}+\frac{\partial w}{\partial z}=0$$

where *u, v*, and *w* are the velocity components in the *X*, *Y*, and *Z* directions, respectively. The melted PCL and composites were assumed to be a laminar flow within the circular needle. All melted materials were considered shear-thinning and non-Newtonian fluids. The CFD simulation was performed by Solidworks 2021 Flow Simulation (Dassault Systèmes, Vélizy-Villacoublay, France) to simulate the extrusion situation and calculate the material extrusion velocity.

The viscosity model dependent on the temperature and the shear rate should be required to solve the above equation. The Williams–Landel–Ferry (WLF) equation was usually used to calculate the viscosity of polymers (Equation (2)),

(2) $$\ln\frac{\eta_{i}}{\eta_{r}}=\frac{-8.86(T-T_{i})}{101.6+T-T_{i}}$$

where *η_i_* is the viscosity of the material at the target temperature and *η_r_* is the material viscosity at the reference temperature. *T* is the current temperature. The reference temperature (*T_r_*) is defined as the glass transition temperature (*T_g_*) + 43 °C. In this study, the Refutas equation [35] using the weight fractions of the materials was employed to determine the viscosity of PCL-based composites (Equations (3)–(5)),

(3) $$\eta =exp\left[exp\left(\frac{A-10.975}{14.534}\right)\right]-0.8,$$

(4) $$A=x_{1}A_{1}+x_{2}A_{2},$$

and

(5) $$A_{i}=14.534\cdot \ln\left[\ln\left(\eta_{i}+0.8\right)\right]+10.975\;(i=1,2)$$

where *η* is the viscosity of the composite, *A* is the composite viscosity blending index, *A_i_* is the average viscosity blending index, and *x_i_* is the weight fraction. The density of the composites was calculated by the rule of mixtures at the printing temperatures using the weight fraction of the materials, and the density of PCL and DMSO_2_ at the printing temperatures was calculated using the coefficient of temperature expansion.

#### 2.3.2. Stacking Model

**Figure 2:**
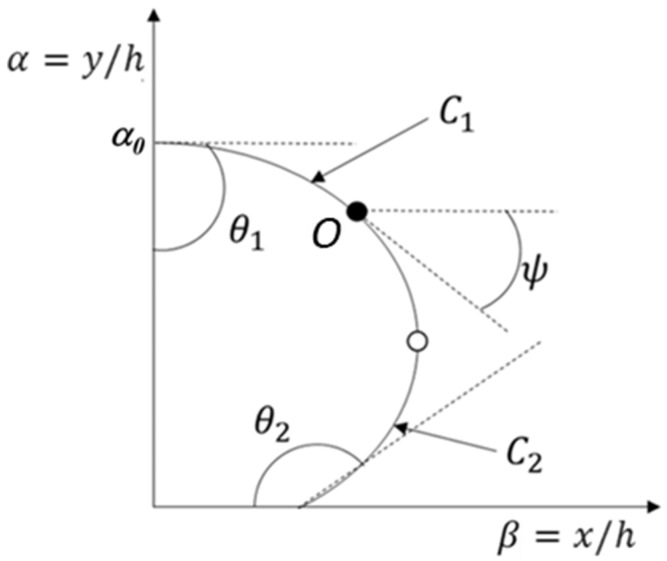
Stacking profile defined by the angle *ψ* and curvatures *C*_1_ and *C*_2_.

The stacking profile of the strut cross-section was assumed a two-dimensional surface energy model on the printing substrate (Figure 2). The cross-section of the printed strut was assumed a static equilibrium shape having two curvatures.

The stacking profile was divided into two curvatures, *C*_1_ and *C*_2_, by the angle *ψ* (*C*_1_*: −π/*2 *≤ ψ ≤ 0, C*_2_*: 0 ≤ ψ ≤ π/*2), and the angle *ψ* on point *O* is the angle between the tangent line of the curvature and the *X* axis on the profile. In the model, the first angle (*θ*_1_) was set as a constant (90°) because the profile was considered symmetric with respect to the *Y* axis, and the second angle (*θ*_2_) was measured from the wettability test [20]. The layer height, the nozzle inner diameter, and the nozzle moving speed were used as fixed parameters during the stacking process. The strut cross-section area should be determined to calculate the strut height and the contact width on the substrate. The area was calculated using the simulated extrusion velocity at the specified printing conditions.

The equilibrium state of a differentially small element can be explained by the connection between pressure variation and curvature, which is already well known as the following equation,

(6) $$P_{a}-P=\gamma \kappa$$

where *P_a_* is the atmospheric pressure, *P* is the internal pressure, *γ* is the surface tension, and the *κ* is the curvature of the strut. In the static equilibrium, it can be deduced that the internal pressure variation conforms to hydrostatic conditions. Therefore, the internal pressure can be defined as

(7) $$P=P_{0}-\rho gh$$

where *P*_0_ is the pressure on the substrate (*h =* 0), the *ρ* is the density of the material, *g* is the gravitational acceleration, and the *h* is the final height of the strut (*h = α*_0_). The density of the composites was calculated using the rule of mixtures, considering the temperature-dependent coefficients of thermal expansion for both PCL and DMSO_2_. Here, the dimensionless coordinates of *α* and *β* were introduced to calculate the stacking profile. The curvature at the point *O* can be expressed with the dimensionless coordinates by

(8) $$\kappa =\frac{1}{\alpha }\frac{\beta^{\prime \prime }}{{\left[1+{\left(\beta^{\prime }\right)}^{2}\right]}^{3/2}}$$

Combining the initial assumptions and the above equilibrium equation, the equation for the stacking profile can be expressed by two elliptic integrals as follows [36]:

(9) $$\beta \left(\alpha \right)=\sqrt{-\frac{\left(1+f\right)}{Hf}}\left[\frac{1}{1+f}\int_{0}^{\frac{\pi -\Omega \left(\alpha \right)}{2}}\frac{d\alpha }{\sqrt{1-J^{2}{sin}^{2}\left(\frac{\pi -\Omega \left(\alpha \right)}{2}\right)}}-\int_{0}^{\frac{\pi -\Omega \left(\alpha \right)}{2}}\sqrt{1-J^{2}{sin}^{2}\left(\frac{\pi -\Omega \left(\alpha \right)}{2}\right)}d\alpha \right]$$

where

(10) $$H=\frac{\rho gh^{2}}{2\gamma }$$

(11) $$f=\frac{4H}{H^{2}+2\left(\sin\theta_{1}+\cos\theta_{2}\right)H+{\left(\sin\theta_{1}-\cos\theta_{2}\right)}^{2}},$$

(12) $$J={\left(\frac{2H}{1+H}\right)}^{1.2},$$

and

(13) $$\Omega \left(\alpha \right)={cos}^{-1}\left[\left(-H\alpha^{2}+\left(\cos\theta_{2}-\sin\theta_{1}-H\right)\alpha -\cos\theta_{2}\right)\right].$$

## 3. Results and Discussion

### 3.1. Numerical Analysis for Extrusion Velocity

**Figure 3:**
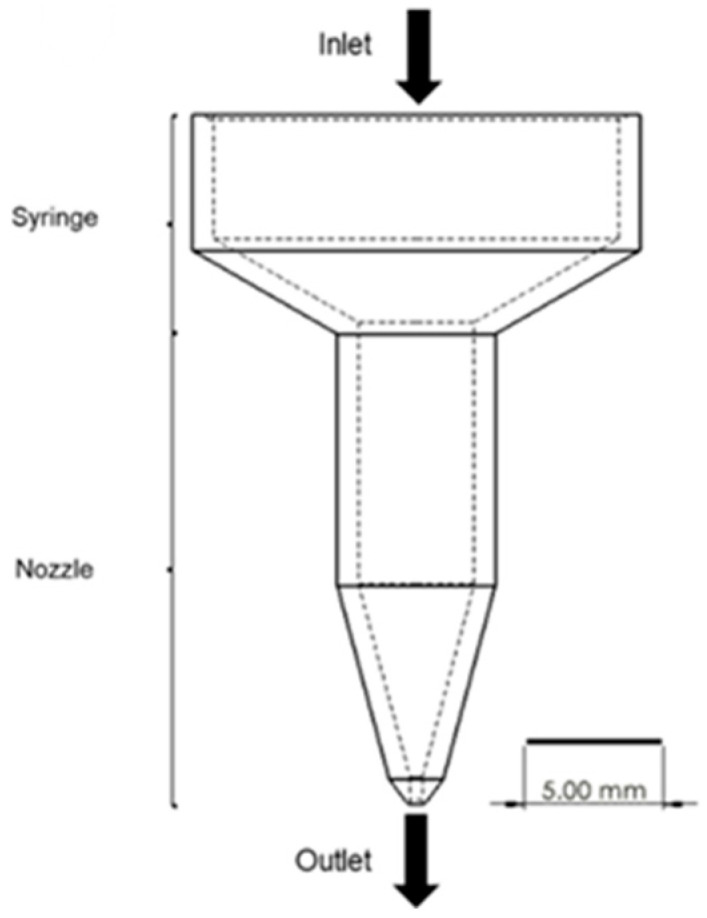
Designed printing system for the model.

The geometry of the material extrusion system was designed to have united the cylinder and the nozzle even though the 3D printer has a separated heating cylinder and the extrusion nozzle (Figure 3). The input temperatures and pressures were the same with the printing conditions for this analysis. The viscosity of PCL and the composites at the reference temperature (120 °C) was referenced in the previous study [37] and calculated by the WLF equation (Equation (2)) for the other desired printing temperatures (Table 2). The initial and boundary conditions were reflected in all printing conditions of this study.

The CFD simulation from SOLIDWORKS Flow Simulation was verified by comparing the experimental material extrusion velocity of PCL and PCL-based composites. SOLIDWORKS Flow Simulation has strong integration with Computer-Aided Design (CAD), ease of use, relatively fast simulation times, and sufficient accuracy for the scope of our study [38]. SOLIDWORKS Flow Simulation employs a discrete numerical method based on the finite volume technique to solve the governing equations. It utilizes a Cartesian rectangular coordinate system. In terms of spatial discretization, a Cartesian grid oriented along the axes is utilized at a considerable distance from the geometric boundary. As a result, the control volumes, resembling mesh cells, take on the shape of rectangular parallelepipeds. When nearing the geometric boundary, a Cartesian cut cell approach is employed. In this approach, the mesh close to the boundary is derived from the original background Cartesian mesh by trimming the original parallelepiped cells that intersect the geometry. Consequently, the near-boundary cells take on the form of polyhedrons with both axis-oriented and arbitrarily oriented plane faces. Although Cartesian grids have limitations in capturing curved or complex geometries, they were sufficient for the relatively smooth nozzle used in this study. Local mesh refinement minimized numerical errors, but more intricate designs may require body-fitted meshes or alternative solvers for improved accuracy [39]. This approach allows SOLIDWORKS Flow Simulation to blend the advantages of methods using regular grids with those offering highly accurate representations of geometric boundaries.

**Figure 4:**
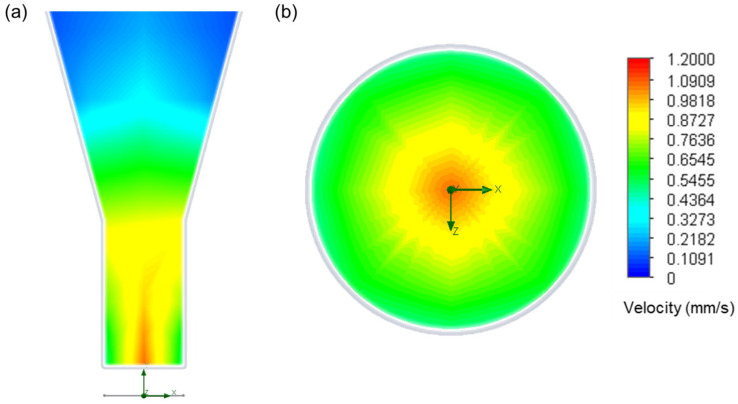
Velocity distribution on (**a**) the cross-section and (**b**) the outlet face at 120 °C and 345 kPa.

The results from the simulation were observed on the cross-section and the outlet velocity distribution, and the extrusion velocity was calculated by average velocity on the outlet face (Figure 4).

**Figure 5:**
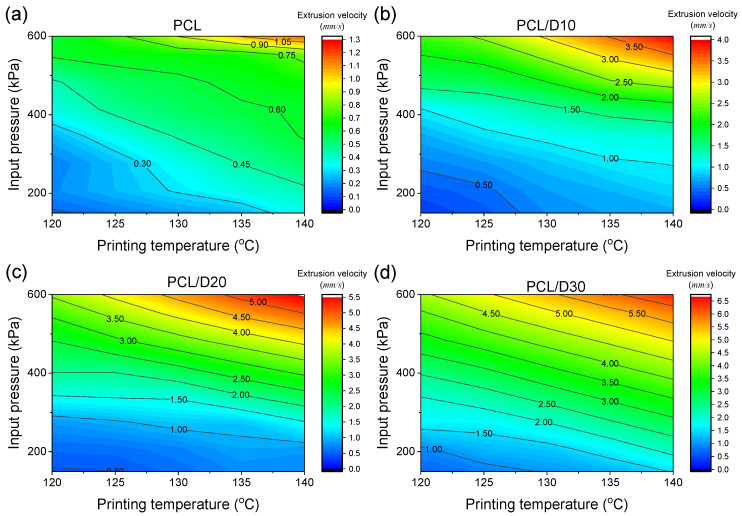
Contour plots for the extrusion velocity of (**a**) PCL, (**b**) PCL/D10, (**c**) PCL/D30, and (**d**) PCL/D30.

The simulated PCL extrusion velocity ranged from 0.130 to 1.339 mm/s while PCL-based composites demonstrated a wider range of 0.202–4.178, 0.451–4.814, and 0.653–6.637 mm/s, in order of DMSO_2_ concentration. The minimum velocity of each material was observed at 120 °C and 138 kPa of the printing conditions in this test, while the maximum velocity was seen at 140 °C and 621 kPa of the printing conditions. The trend in velocity can be attributed to the linear relationship observed when the printing conditions, such as temperature and pressure, were changed. Notably, the high concentration of DMSO_2_ in the composites allowed for a broader range of velocities to be extruded at the same printing conditions compared to pure PCL, as shown in the contour plot in Figure 5.

**Figure 6:**
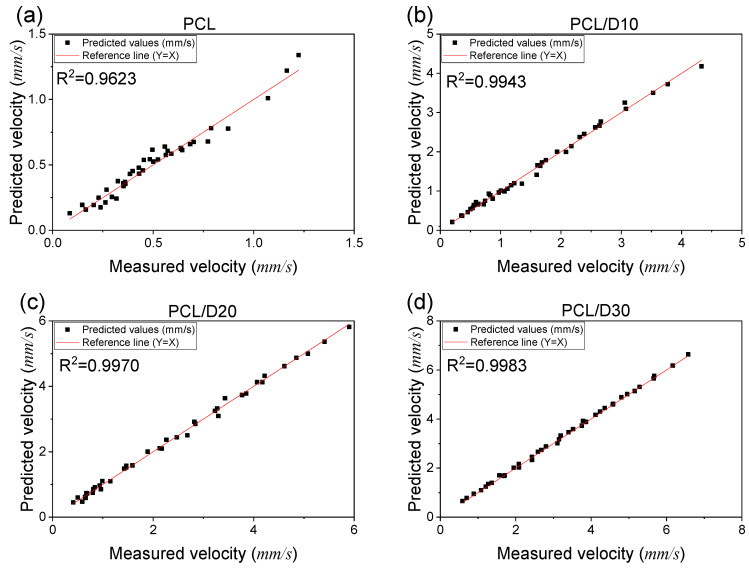
Correlation between the measured and the predicted extrusion velocity: (**a**) PCL, (**b**) PCL/D10, (**c**) PCL/D30, and (**d**) PCL/D30.

This indicates that high viscous materials have low sensitivity to changes in printing temperature and pressure. The CFD analysis was able to accurately predict the extrusion velocity based on printing conditions, with R^2^ values of 0.9623, 0.9943, 0.9970, and 0.9983 for PCL, PCL/D10, PCL/D20, and PCL/D30, respectively (Figure 6).

### 3.2. Numerical Analysis for Strut Diameter

**Figure 7:**
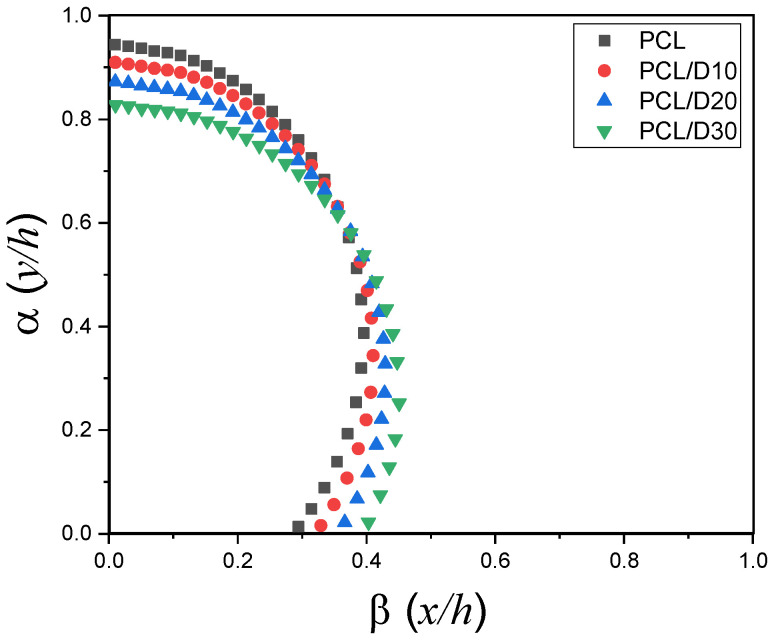
Stacking profile for the pure PCL and PCL composites at 120 °C and 345 kPa.

**Figure 8:**
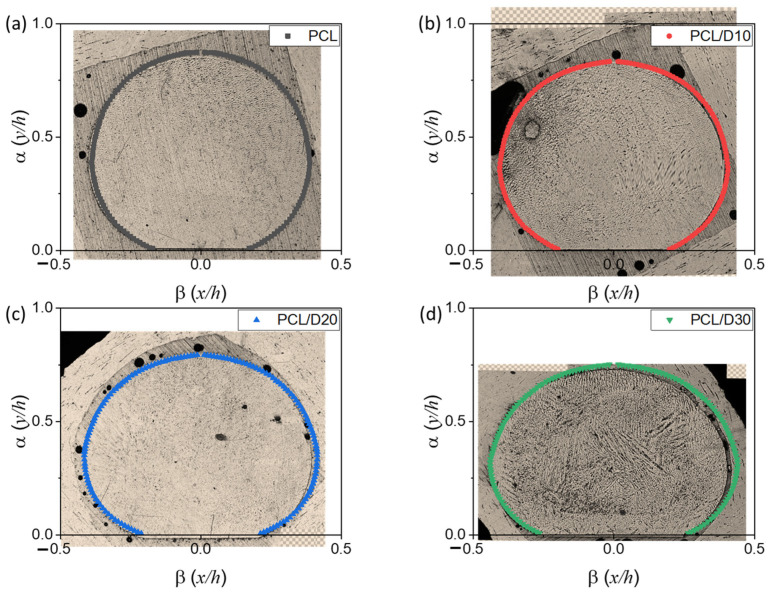
Cross-sectional images with the stacking profile at 120 °C and 345 kPa of (**a**) PCL, (**b**) PCL/D10, (**c**) PCL/D20, and (**d**) PCL/D30.

The stacking profile at 120 °C and 345 kPa was illustrated in Figure 7 and the cross-sectional image merged with the stacking profile was displayed in Figure 8. The study demonstrated that higher concentrations of DMSO_2_ resulted in a reduced strut height. This implies that the addition of DMSO_2_ influenced the vertical strut dimensions, leading to a decrease in the overall height of the 3D bio-scaffold. In contrast, the contact length with the substrate and strut diameter showed an increase as the concentration of DMSO_2_ increased, implying that DMSO_2_ influenced the horizontal dimensions and contact characteristics of the printed struts.

**Figure 9:**
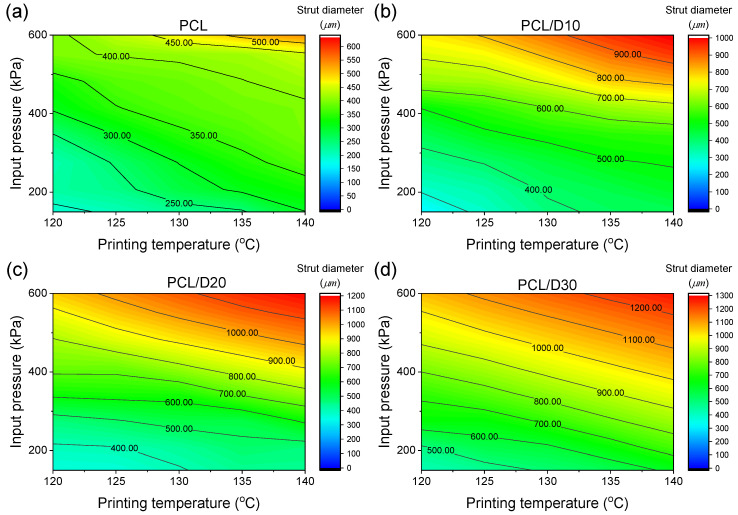
Contour plots for the strut diameter of (**a**) PCL, (**b**) PCL/D10, (**c**) PCL/D30, and (**d**) PCL/D30.

At 120 °C and 345 kPa of the printing conditions, the strut diameter of pure PCL was calculated to be 181.25 µm. Increasing the DMSO_2_ concentration led to a significant increase in strut diameter, with values increasing by 27.95, 86.41, and 124.24% for PCL/D10, PCL/D20, and PCL/D30, respectively. At 140 °C and 621 kPa of the printing conditions, the strut diameter of pure PCL was 582.25 µm, while the composites exhibited even larger strut diameters, with values of 1028.33, 1213.17, and 1296.13 µm for PCL/D10, PCL/D20, and PCL/D30, respectively. This showed a similar trend to the extrusion velocity model in which the results increased with increasing printing temperature and pressure. The contour plot in Figure 9 illustrated the calculated strut diameter for the composites.

**Figure 10:**
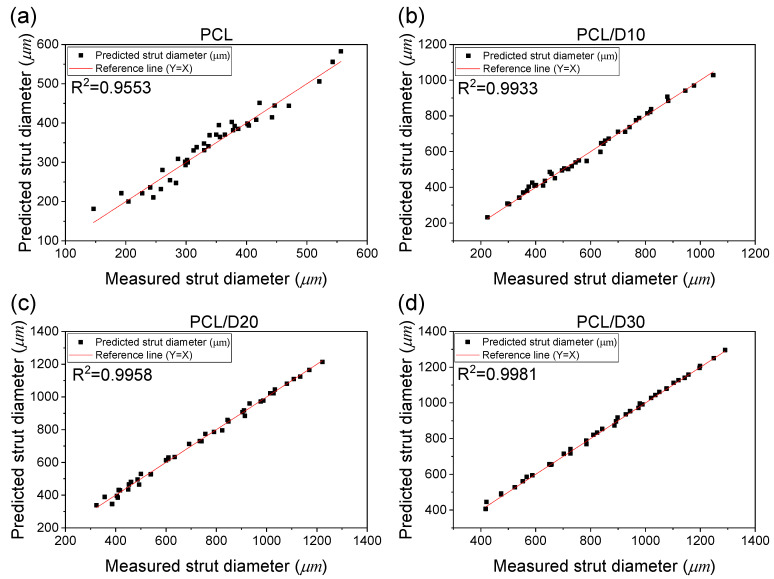
Correlation between the measured and predicted strut diameter: (**a**) PCL, (**b**) PCL/D10, (**c**) PCL/D30, and (**d**) PCL/D30.

The evaluation of the developed stacking model using R^2^ values for the different PCL-based composites demonstrates the accuracy and reliability of the model in predicting the strut diameter. In this case, the obtained R^2^ values of 0.9553, 0.9933, 0.9958, and 0.9981 for PCL, PCL/D10, PCL/D20, and PCL/D30, respectively, indicated a high level of accuracy in predicting the strut diameter for these composites (Figure 10). These values suggested that the developed stacking model closely approximated the experimental data, with a strong correlation between the predicted and actual values of the strut diameter.

### 3.3. Printing Condition Effect on the Extrusion Velocity and Strut Diameter

The gradient of the printing temperature and pressure can represent how changes in these printing conditions affect the extrusion velocity and strut diameter in material extrusion 3D printing method. It was found that an increase of 1 °C in temperature resulted in a 23.3% increase in the extrusion velocity. Similarly, an increase of 100 kPa in pressure led to a significant 19.1% increase in the extrusion velocity. This suggested a positive relationship between the printing conditions and extrusion velocity, indicating that higher temperatures facilitate faster material flow and deposition during printing. Regarding the strut diameter, an increase of 1 °C in temperature corresponded to a 21.6% increase in the strut diameter. In addition, an increase of 100 kPa in pressure resulted in a 16.6% increase in the strut diameter.

This indicates that a change in temperature allows for a notable increase in extrusion velocity, resulting in a thicker strut diameter. The printing conditions, including temperature and pressure, are directly connected to the printing process, and they play a crucial role in determining scaffold quality. Manipulating the printing conditions, such as temperature and pressure, is essential for optimizing the entire printing process and achieving the desired scaffold characteristics. By adjusting the printing conditions, it can obtain the desired resolution and accuracy of the strut, as well as control the scaffold volume and pore size by designing the total volume and rec of the 3D scaffold.

### 3.4. Material Property Effect on the Extrusion Velocity and Strut Diameter

**Figure 11:**
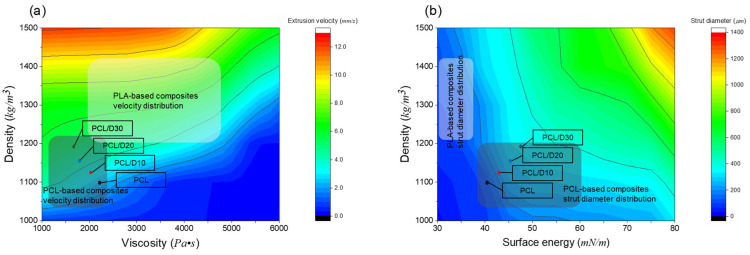
Contour plot of the material property effect for (**a**) the extrusion velocity and (**b**) the strut diameter.

The developed numerical models in this study can be employed to investigate the material property effect on the extrusion velocity and strut diameter by changing specific material properties. The density and viscosity of material can affect the extrusion velocity directly, and surface energy, as well as density, can influence the strut diameter. When the printing conditions were assumed to be 120 °C of the printing temperature and 345 kPa of printing pressure, the model generated contour plots to depict the effects of the density and viscosity on the extrusion velocity (Figure 11a). The first contour plot displayed the extrusion velocity range of 0.56–12.5 mm/s in the density ranging from 1000 to 1500 kg/m^3^ and viscosity of 1000–6000 Pa·s. The second contour plot revealed the strut diameter range of 25–1250 µm in the density ranging from 1000–1500 kg/m^3^ and the surface energy of 30–80 mN/m (Figure 11b).

In the case of the extrusion velocity, it suggested a negative relationship between the extrusion velocity and viscosity and a positive relationship between the extrusion velocity and density. This means that the 3D printing process time can be reduced with the biomaterial having high density and low viscosity. In terms of the strut diameter, it was found that increasing the surface energy and density of the biomaterial resulted in an increase in the strut diameter. This indicates that a selection of biomaterial properties can change the dimension of the 3D bio-scaffold at the same printing conditions.

The extrusion velocity and strut diameter of PCL and composites at 120 °C and 345 kPa were expressed as dots in the contour plots (Figure 10), and the range of the extrusion velocity and strut diameter of polylactic acid (PLA)-based composites and PCL-based composites were also displayed in the plots. The composite with high concentrations of DMSO_2_ showed faster extrusion velocity due to the high density and low viscosity compared to the pure PCL. Moreover, the strut diameter was increased with DMSO_2_ concentration because DMSO_2_ has higher surface energy than the pure PCL. The material properties of PLA composites were researched to have higher density, viscosity, and lower surface energy than PCL-based composites [40,41,42]. Consequently, PLA composites were predicted to exhibit a wider range of extrusion velocities and narrower strut diameters.

These findings provide valuable insights into the relationships between material properties and the extrusion velocity and strut diameter in different types of composites used in 3D printing. They contribute to understanding the behavior of different materials and assist in the selection of appropriate printing parameters to achieve desired extrusion velocities and strut diameters for 3D bio-scaffold fabrication.

### 3.5. 3D Scaffold Fabrication Under Optimal Printing Conditionsr

**Figure 12:**
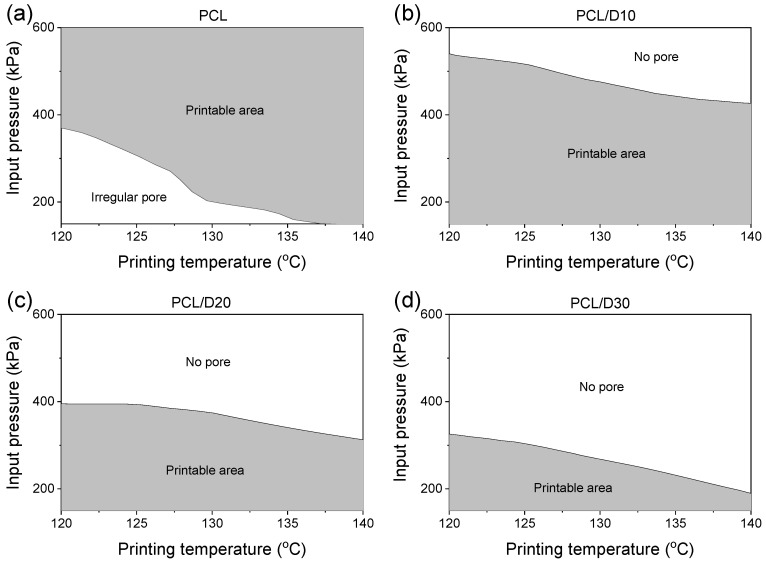
The printable area under the printing conditions of (**a**) PCL, (**b**) PCL/D10, (**c**) PCL/D20, and (**d**) PCL/D30.

The models can help the optimization of the printing conditions to fabricate the 3D scaffold when the nozzle moving speed (0.8 mm/s) and the strut pitch (700 µm) were fixed. The extrusion velocity should be fast enough to form a linear line, and the strut diameter should be thinner than the designed strut pitch to create pores. The PCL and composites were printed in the shape of wavy lines when the extrusion velocity was less than 0.21 mm/s [37]. The printable area under printing condition ranges— temperature in the range of 120–140 °C and pressure in the range of 150–600 kPa—was set as the extrusion velocity formed the straight line and with the strut diameter not exceeding the designed strut pitch. Therefore, the printable area represents the combination of temperature and pressure conditions. The underline indicated the minimum extrusion velocity to print a linear line, and the upper line meant that the printed strut diameter was the same as the designed strut pitch (Figure 12).

The strut diameter of PCL was thinner than the designed strut pitch at all printing conditions, while the extrusion velocity was not enough to print the linear line at low printing temperature and pressure. On the other hand, all PCL and DMSO_2_ composites showed a suitable extrusion velocity to not print the wavy line, but the strut diameter exceeded the designed strut pitch at high pressure.

These combining printing conditions are crucial for obtaining a well-fabricated 3D scaffold with the desired characteristics. The printable area study provided valuable information regarding the range of temperature and pressure conditions that were employed to form the linear line and interconnected pores in the 3D scaffold even though the nozzle moving speed and designed strut pitch were restricted. The information can help to optimize the printing conditions during the printing process and achieve the desired 3D scaffold characteristics for various printing conditions.

## 4. Conclusions

In this study, a unified numerical model was developed to accurately predict the extrusion velocity and strut diameter of PCL and DMSO_2_ composites, aiming to predict the strut geometry. The research conducted in this study resulted in precise predictions for the extrusion velocity and strut diameter based on the 3D printing conditions and material properties.

The printing conditions for the model included a temperature range of 120–140 °C and a pressure range of 138–621 kPa. In the analysis, the nozzle moving speed and designed strut pitch were set to 0.8 mm/s and 700 µm, respectively. At a printing temperature of 120 °C and a printing pressure of 138 kPa, all materials exhibited the slowest extrusion velocity and the thinnest strut diameter. Conversely, the fastest extrusion velocity and the thickest strut diameter were observed at a printing temperature of 140 °C and a printing pressure of 621 kPa. The developed numerical models provided high accuracy, as evidenced by R^2^ values exceeding 0.95 for both the extrusion velocity and strut diameter predictions.

The extrusion velocity exhibited a 23.3% increase per 1 °C rise in temperature and a 19.1% increase per 100 kPa rise in pressure, and the strut diameter experienced a 21.6% increase per 1 °C rise in temperature and a 16.6% increase per 100 kPa rise in pressure. Moreover, the addition of DMSO_2_ to the PCL matrix increased the extrusion velocity due to the low viscosity of DMSO_2_. It also expanded the strut diameter in the horizontal direction, which can be attributed to the high surface energy of DMSO_2_. Additionally, the geometry of the strut cross-section was changed. This model can also determine the printable range of pore formation conditions. The extrusion velocity should be fast enough to maintain a linear flow, while the strut diameter should be smaller than the designed strut pitch to create pores.

In summary, the implementation of the unified numerical models offers significant practical implications for biomaterial selection and optimization of the 3D printing process. The precise prediction of the strut geometry from the extrusion velocity and strut diameter informed decision-making when it comes to selecting appropriate biomaterials and fine-tuning printing parameters. This approach facilitates the fabrication of 3D bio-scaffolds with desirable structural characteristics. Furthermore, this modeling framework might be extended to more complex scenarios, such as cell-laden bioinks or temperature-sensitive phase change materials, thereby broadening its applicability in functional tissue engineering.

## Figures and Tables

**Table 1:** Material properties of PCL and DMSO_2_.

Material	Appearance	Molecular Weight (g/mol)	Density (kg/m^3^)	Viscosity @120 °C (Pa∙s)	CTE * (10^−6^/°C)	Surface Tension (N/m)
PCL	Powder	50,000	1145	2211.35	165	0.040
DMSO_2_	Powder	94.13	1450	0.00114	88	0.060

* Coefficient of temperature expansion.

**Table 2:** Calculated shear viscosity of PCL and composites (unit: Pa·s).

Temp. (°C)	PCL	PCL/D10	PCL/D20	PCL/D30
120	1286.6	863.0	696.9	333.8
125	1094.4	816.3	662.1	316.6
130	1018.9	762.7	622.2	286.5
135	946.0	749.7	577.1	251.1
140	889.5	720.1	526.9	229.1

## Data Availability

The original contributions presented in the study are included in the article, further inquiries can be directed to the corresponding author.
